# Forgotten but not gone: Yersinia infections in England, 1975 to 2020

**DOI:** 10.2807/1560-7917.ES.2023.28.14.2200516

**Published:** 2023-04-06

**Authors:** Dana Šumilo, Nicola K Love, Rohini Manuel, Girija Dabke, Karthik Paranthaman, Claire Jenkins, Noel D McCarthy

**Affiliations:** 1 Division of Health Sciences, University of Warwick, Coventry, United Kingdom; 2 National Institute for Health and Care Research Health Protection Research Unit in Gastrointestinal Infections at University of Liverpool, Liverpool, United Kingdom; 3 Health Protection Operations, UK Health Security Agency, London, United Kingdom; 4 Science, UK Health Security Agency, London, United Kingdom; 5 Health Protection Operations, UK Health Security Agency, South East, Fareham, United Kingdom; 6 Institute of Population Health, Trinity College Dublin, University of Dublin, Dublin, Ireland

**Keywords:** Yersinia Enterocolitica, England, Epidemiology, Incidence, Surveillance

## Abstract

**Background:**

Yersiniosis is one of the most common food-borne zoonoses in Europe, but there are large variations in the reported incidence between different countries.

**Aim:**

We aimed to describe the trends and epidemiology of laboratory-confirmed *Yersinia* infections in England and estimate the average annual number of undiagnosed *Yersinia enterocolitica* cases, accounting for under-ascertainment.

**Methods:**

We analysed national surveillance data on *Yersinia* cases reported by laboratories in England between 1975 and 2020 and enhanced surveillance questionnaires from patients diagnosed in a laboratory that has implemented routine *Yersinia* testing of diarrhoeic samples since 2016.

**Results:**

The highest incidence of *Yersinia* infections in England (1.4 cases per 100,000 population) was recorded in 1988 and 1989, with *Y. enterocolitica* being the predominant species. The reported incidence of *Yersinia* infections declined during the 1990s and remained low until 2016. Following introduction of commercial PCR at a single laboratory in the South East, the annual incidence increased markedly (13.6 cases per 100,000 population in the catchment area between 2017 and 2020). There were notable changes in age and seasonal distribution of cases over time. The majority of infections were not linked to foreign travel and one in five patients was admitted to hospital. We estimate that around 7,500 *Y. enterocolitica* infections may be undiagnosed in England annually.

**Conclusions:**

Findings suggest a considerable number of undiagnosed yersiniosis cases in England, with possibly important changes in the epidemiology. The apparently low incidence of yersiniosis in England is probably due to limited laboratory testing.

**Key public health message**

**What did you want to address in this study?**

Yersiniosis is a bacterial infection and one of the most common causes of food poisoning in Europe. The reported numbers of cases vary markedly between countries and in England are relatively low. We examined data on *Yersinia* cases in England between 1975 and 2020 to describe trends over time and to estimate the current number of undiagnosed *Y. enterocolitica* cases, accounting for limited laboratory testing for this infection in England.

**What have we learnt from this study?**

Our findings suggest a considerable number of undiagnosed yersiniosis cases in England, with higher incidence than reported in other countries in Europe. There are also marked changes over time and differences in seasonal and age distribution of cases. This changing epidemiology may reflect changing sources of infection and the need for surveillance and further investigation to guide more accurate control interventions.

**What are the implications of your findings for public health?**

The apparently low overall number of reported yersiniosis cases in England is probably due to lack of laboratory testing. This is also likely to be the case in some other low-incidence countries across Europe. The increasing use of PCR offers an opportunity to investigate and control this infection more fully.

## Introduction

Yersiniosis, most often caused by *Yersinia enterocolitica,* is one of the most common bacterial food-borne zoonoses in Europe with reported overall incidence of 1.8 cases per 100,000 population in 2020 [1]. There is, however, marked variation among countries, with the highest numbers of cases per 100,000 population reported in Denmark and Finland (7.1 and 7.0, respectively) and the lowest in Romania and Bulgaria (0.03 and 0.06, respectively) [1]. Transmission is primarily faecal–oral via food or water contaminated with animal faeces [2]. Yersiniosis has been associated with the consumption of pork meat (raw or undercooked), occupational exposure to pigs, untreated drinking water, milk, vegetables, juices, ready-to-eat and other foods [3-6]. The incidence of yersiniosis in Europe is higher in males and in children under 5 years, and no clear seasonal pattern has been reported over the last decade [1,3]. Yersiniosis commonly presents as diarrhoea, abdominal pain and fever, and can manifest as acute mesenteric lymphadenitis and terminal ileitis. Although it is usually self-limiting with a low case fatality rate (0.05%), symptoms often persist for several weeks [3,6].

The reported incidence of *Yersinia* infections in the United Kingdom (UK) is well below the European average (0.2 cases per 100,000 in 2019) [3]. Routine testing for *Yersinia* is not currently recommended in the UK, unless there is a clinical suspicion (e.g. appendicitis, mesenteric lymphadenitis, terminal ileitis or reactive arthritis) [7]. The aim of this study was to describe the changing incidence and epidemiology of diagnosed *Yersinia* infections in England between 1975 and 2020 and to estimate the potential under-ascertainment of *Y. enterocolitica* due to the lack of routine testing.

## Methods

### Data source

All diagnostic laboratories in England submit reports of confirmed infectious pathogens to the UK Health Security Agency (UKHSA) and are stored in a national surveillance database called the Second Generation Surveillance System (SGSS) [8]. Information on patient demographics (age, sex, region of residence) and specimen details (specimen date, type, referral, test method, laboratory, organism species) for all *Yersinia* cases (excluding *Yersinia pestis)* reported from 1975 until 2020, inclusive, was extracted from SGSS. Data were analysed by episode (episode length in SGSS is defined as 14 days with repeated specimens deduplicated across this period) [8], with later repeat positive results included as a new episode (estimated to be ca 2%) [8,9].

### Descriptive analysis 

The annual and regional incidences of *Yersinia* per 100,000 population were determined using the Office for National Statistics (ONS) mid-year population estimates for respective geographies for each calendar year [10]. We undertook descriptive analysis to summarise the characteristics of infections comparing the time periods when the reported incidence was high (1987–1993, with culture traditionally used for *Yersinia* detection and routine testing occurring in many laboratories), low (2000–2015, with culture traditionally used for *Yersinia* detection and guidelines advising testing only on clinical request) and increasing (2017–2020), due to one diagnostic laboratory, the Portsmouth Hospitals NHS Trust microbiology laboratory (Portsmouth laboratory) in Hampshire in the South East of England implementing routine PCR testing of diarrhoeic samples for *Yersinia *as part of a multiplex PCR assay [9]. For the time periods of high and increasing incidence, we also identified laboratories contributing the largest number of *Y. enterocolitica* diagnoses. The numbers of total tests conducted by different laboratories are not recorded and reported, as SGSS only records positive results, and the estimation of the proportion of positive samples was not possible. We compared the characteristics of cases diagnosed by laboratories reporting high numbers with cases identified by other laboratories, to assess if the practices of these hospital laboratories or their catchment populations may have affected the overall characteristics of cases during these time periods. Chi-squared test was used to compare differences in categorical data.

### Estimating undiagnosed *Yersinia enterocolitica* infections

The potential average number of undiagnosed *Y. enterocolitica* infections annually (2017–2020) in England was estimated by extrapolating the incidence reported by the Portsmouth laboratory using their reported cases and the estimated hospital catchment population as a denominator [11]. We also estimated the incidence of *Y. enterocolitica* in the catchment areas of laboratories that contributed the largest number of cases in the period of previously high incidence in England (1987–1993). Since no historical catchment population estimates for the laboratories were available, the ONS average population estimates for 1983 to 1997 in the laboratory’s local authority where the cases resided were used as the denominator.

### Analysis of enhanced surveillance data

We also summarised data from enhanced surveillance questionnaires completed by patients with positive PCR results for *Yersinia* reported from the Portsmouth laboratory in the South East of England between September 2018 and March 2020. The questionnaire collected information on demographic details, symptoms, use of healthcare services, travel history, consumption of food and water, and animal contact in the 7 days before the illness.

## Results

**Figure 1:**
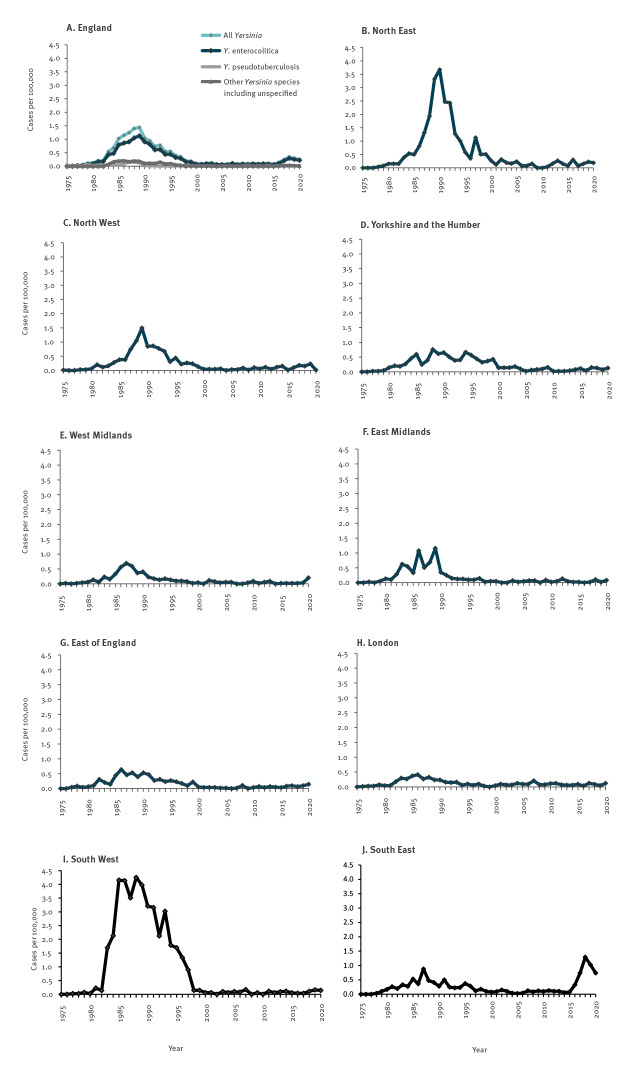
Incidence of reported infections with different *Yersinia* species in England (n = 8,023) and *Y. enterocolitica* by region (n = 6,397), 1975–2020

Overall, 8,023 laboratory-confirmed cases of *Yersinia* infection were recorded in England between 1975 and 2020. Cases increased sharply during the 1980s to a peak of 1.4 cases per 100,000 population in 1988 and 1989, followed by a steep decrease (Figure 1A). The incidence remained very low (0.1 cases per 100,000 population) until 2016, increasing to 0.2–0.3 cases per 100,000 population in the following years.

 The large majority (79.7%; 6,397/8,023) of infections during the period 1975 to 2020 were caused by *Y. enterocolitica*. *Yersinia frederiksenii* was detected in 9.5% (764/8,023), *Y. pseudotuberculosis* in 7.1% (573/8,023), other *Yersinia* species were identified in 1.9% (151/8,023) and for the remaining 1.7% (138/8,023) the *Yersinia* species was not specified in the records. From 2017 to 2020, 87.9% (546/621) of cases were *Y. enterocolitica*, 4.2% (26/621) were *Y. pseudotuberculosis,* 2.3% (14/621) were *Y. frederiksenii*, 1.1% (7/621) were other *Yersinia* species and in 4.5% (28/621) *Yersinia* species was not specified. The Table summarises the characteristics of cases of yersiniosis caused by *Y. enterocolitica* from 1983 to 1997 when the incidence was high, 2000 to 2015 when the incidence was very low and 2017 to 2020 when an increased incidence was observed in association with one laboratory (the Portsmouth laboratory) in the South East of England implementing routine PCR testing.

**Table:** Characteristics of *Yersinia enterocolitica* cases during three different time periods, England, 1975–2020 (n = 6,397)

Characteristics	Time period							
	1983–1997 (high incidence)		2000–2015 (low incidence)		2017–2020 (PCR testing)		Total (1975–2020)	
	n	%	n	%	n	%	n	%
**Sex**								
Male	2,351	48.9	252	42.9	283	51.8	3,106	48.6
Female	2,389	49.7	270	45.9	258	47.3	3,151	49.3
Unknown	63	1.3	66	11.2	5	0.9	140	2.2
**Age (years)**								
0–4	773	16.1	47	8.0	33	6.0	914	14.3
5–14	425	8.8	44	7.5	34	6.2	540	8.4
15–24	579	12.1	31	5.3	51	9.3	737	11.5
25–44	1,149	23.9	121	20.6	122	22.3	1,512	23.6
45–64	658	13.7	114	19.4	139	25.5	979	15.3
≥ 65	634	13.2	163	27.7	164	30.0	1,023	16.0
Unknown	585	12.2	68	11.6	3	0.5	692	10.8
**Region**								
North West	606	12.6	66	11.2	42	7.7	777	12.1
North East	562	11.7	59	10.0	17	3.1	687	10.7
West Midlands	227	4.7	37	6.3	17	3.1	304	4.8
Yorkshire and the Humber	357	7.4	71	12.1	26	4.8	525	8.2
East Midlands	247	5.1	31	5.3	12	2.2	316	4.9
East of England	269	5.6	35	6.0	25	4.6	382	6.0
South West	1,902	39.6	58	9.9	25	4.6	2023	31.6
London	217	4.5	114	19.4	34	6.2	398	6.2
South East	416	8.7	117	19.9	348	63.7	985	15.4
**Month of diagnosis**								
January	365	7.6	47	8.0	38	7.0	486	7.6
February	327	6.8	44	7.5	45	8.2	439	6.9
March	356	7.4	59	10.0	37	6.8	486	7.6
April	369	7.7	48	8.2	50	9.2	499	7.8
May	413	8.6	54	9.2	81	14.8	587	9.2
June	450	9.4	62	10.5	81	14.8	622	9.7
July	478	10.0	44	7.5	52	9.5	623	9.7
August	442	9.2	51	8.7	39	7.1	585	9.1
September	412	8.6	38	6.5	25	4.6	520	8.1
October	467	9.7	34	5.8	35	6.4	573	9.0
November	413	8.6	64	10.9	28	5.1	550	8.6
December	311	6.5	43	7.3	35	6.4	427	6.7
**Specimen type**								
Faeces/stool/lower gastrointestinal tract	4,146	86.3	408	69.4	459	84.1	5,324	83.2
Blood/ serum	396	8.2	133	22.6	40	7.3	622	9.7
Other specimen	28	0.6	44	7.5	41	7.5	136	2.1
Unknown	233	4.9	3	0.5	6	1.1	315	4.9
**Test method**								
Culture	2,277	47.4	479	81.5	296	54.2	3,252	50.8
Genomic/PCR/LCR detection	0	0	0	0	206	37.7	220	3.4
Other technique	169	3.5	20	3.4	11	2.0	216	3.4
Unknown^a^	2,357	49.1	89	15.1	33	6.0	2,709	42.3
**Sample referral**								
Hospital inpatient	0	0	119	20.2	97	17.8	229	3.6
Other	0	0	194	33.0	394	72.2	628	9.8
Not recorded	4,803	100	275	46.8	55	10.1	5,540	86.6

LCR: ligase chain reaction.

^a^ Test method was completed for the minority of cases until 1990. Between 1990 and 1997, 95.8% (2,053/2,143) of cases were identified using culture, 3.8% (81/2,143) using another technique, and for 0.4% (9/2,143) the method was not recorded.

The reported incidence of *Y. enterocolitica* cases was highest during the 1980s and 1990s. The rates were highest at this time were in the South West and North East (Figure 1B–J). From 1983 to 1997, 86.9% (1,652/1,902) of *Y. enterocolitica* cases in the South West were diagnosed by one of 18 laboratories reporting *Y. enterocolitica* cases in the region, the Poole microbiology laboratory. The *Y. enterocolitica* cases diagnosed at the Poole laboratory accounted for 34.6% (1,660/4,803) of all cases in England in that period. The average annual reported incidence in the direct local authority catchment area of the Poole laboratory reached 32.8 cases per 100,000 population. In the North East, during 1983 to 1997, 43.6% (245/562) of *Y. enterocolitica* cases were reported by the Sunderland Royal Infirmary, one of 16 laboratories reporting *Y. enterocolitica* cases in the region during this time period. The *Y. enterocolitica* cases diagnosed at the Sunderland Royal Infirmary accounted for 5.1% (245/4,803) of all cases in England. The average annual incidence in the direct catchment area of the Sunderland Royal Infirmary during this time period reached 5.5 cases per 100,000 population.

Between 2017 and 2020, most *Y. enterocolitica* cases in the South East (92.0%, 320/348) were identified by the Portsmouth laboratory, one of 11 laboratories that diagnosed *Y. enterocolitica* cases in the South East region during this time period. The *Y. enterocolitica* cases reported by the Portsmouth laboratory accounted for 59.7% (326/546) of all cases reported in England. The average annual incidence of *Y. enterocolitica* infections for the catchment population of the laboratory was 13.6 cases per 100,000 population. If this incidence of *Y. enterocolitica* was consistent across England in the catchment populations of laboratories not undertaking routine PCR testing, an average of 7,500 *Y. enterocolitica* infections were undiagnosed annually during 2017–2020.

**Figure 2:**
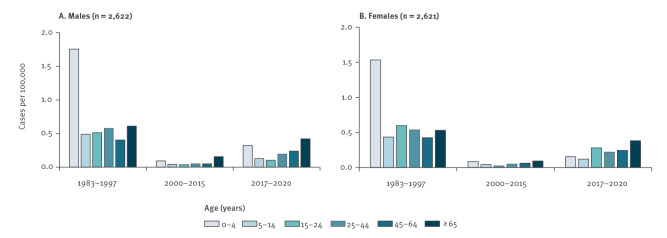
Average annual incidence of *Yersinia enterocolitica* infections by sex and age during three time periods when incidence was the highest (1983–1997) and lowest (2000–2015), and the most recent time period (2017–2020), England (n = 5,243)

During the 1980s and 1990s, the recorded incidence of *Y. enterocolitica* infections in England was substantially higher among children under 5 years than other age groups. This demographic profile of cases changed in the low incidence era and again following the introduction of PCR testing (Figure 2).

**Figure 3:**
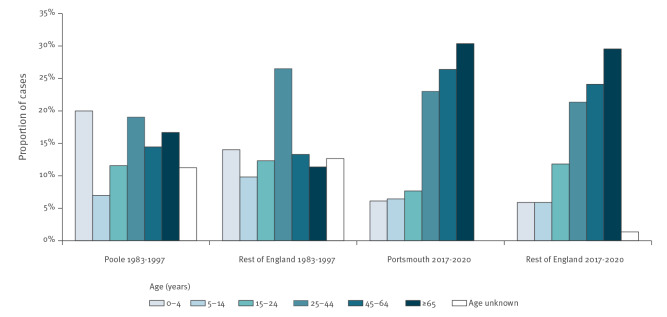
Proportion of *Yersinia enterocolitica* cases by age diagnosed by the Poole microbiology laboratory and the rest of diagnostic laboratories, 1983–1997, and by Portsmouth laboratory and the rest of diagnostic laboratories, 2017–2020, England (n = 5,349)

Figure 3 compares the age distributions of cases reported by laboratories diagnosing much higher incidence than elsewhere in England with the age distributions from all other laboratories in the same time period. Age distributions varied substantially more across time than between these different laboratories and do not suggest that the practices or catchment populations of these hospitals are driving the observed changes in age distribution over time. Almost one third of all cases between 2017 and 2020 diagnosed nationally and in Portsmouth were 65 years or older with the difference between the Portsmouth laboratory (routine testing) and other laboratories (clinically indicated testing) not statistically significant (p = 0.84). However, there was a significant increase in, for example, the proportion of cases 65 years and older diagnosed in the rest of the England between 1983 and 1997 and between 2017 and 2020 (p < 0.0001).

**Figure 4:**
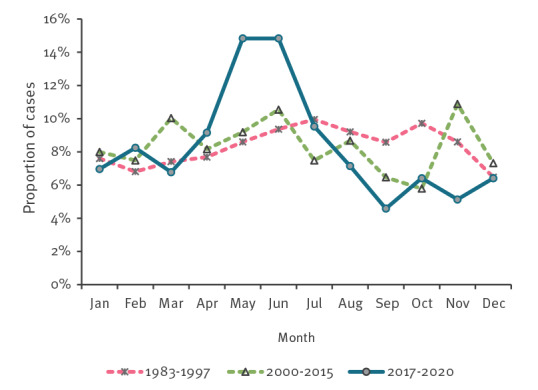
Seasonal distribution of *Yersinia enterocolitica* cases during three different time periods. England, 1983–1997, 2000–2015 and 2017–2020 (n = 5,937)

In the most recent time period 2017 to 2020, but not before, almost one third of cases (29.7%; 162/546) were reported during May and June (Figure 4). Except for the Portsmouth laboratory where PCR was used routinely, culture remained the main technique for detecting reported *Y. enterocolitica* in England.

Enhanced surveillance questionnaires were completed by 39.1% (45/115) patients in the catchment area of the Portsmouth laboratory during 2018 and 2019. The median age of responders was 52 years (interquartile range: 44–70), similar to the median age (51 years) of all patients. Common symptoms were diarrhoea (40/44, one case did not provide symptom details), stomach pain (34/44), nausea (22/44) and headache (22/44). Also reported were joint/back pain (11/44), vomiting (11/44), fever (11/44), muscle pain (11/44), blood in stool (10/44) and dizziness/fainting (9/44). Questionnaire completion was a median of 29 days after symptom onset date where reported (32/45). Around half of the responders (20/39) reported that they were still ill at the time of questionnaire completion. For those who had recovered, the median duration of illness was 16 days. Where hospitalisation status was known, nine of 44 had been admitted to hospital for a median of 2 days.

A large proportion (18/41) reported treatment of their yersiniosis with antibiotics. Over one third of responders (11/40) reported a medical condition affecting their bowel, such as diverticulitis, irritable bowel syndrome or inflammatory bowel disease. Travel or return to the UK from abroad within 7 days before illness was reported by 10 of 43). No contact with pigs was reported but six of 42 reported handling raw pork or gammon in the 7 days before illness.

## Discussion

Reported incidence of *Yersinia* infections in England varied markedly between 1975 and 2020, with the highest incidence reported in the late 1980s and the lowest during the period 2000 to 2015. A recent increase during 2017 to 2020 can be attributed to the implementation of routine PCR at a single hospital laboratory in the South East of England in 2016 [9].

In regions where the incidence was the highest during the 1980s and 1990s (the South West and North East of England), single diagnostic laboratories accounted for a substantial proportion of all cases reported in these regions. The decline in notifications of *Yersinia* from laboratories across England coincided with the introduction of Clinical Pathology Accreditation (CPA), now part of UK Accreditation Service, the national accreditation body for the UK, which was established in 1992 [12]. A CPA laboratory inspection involved an external audit of the ability to provide clinical services that adhered to a defined standard of practice, which was agreed and confirmed by peer review. These UK Standards for Microbiology Investigations (UK SMIs) state that faecal specimens should be tested for *Yersinia* species only where there is a clinical suspicion of yersiniosis [7]. Reporting of diagnosed *Yersinia* infections may also have changed across this time, although there is no evidence for alterations linked to reorganisations in UK public health authorities or reporting guidelines. These strands of evidence all indicate that the current and historically recorded incidence of laboratory-confirmed *Yersinia* infection in England has been substantially driven by variable patterns of testing, and that overall low levels may not reflect the true incidence of infection. Our findings highlight a surveillance gap for yersiniosis in England.

Some real changes in epidemiology may also have occurred over time. The proportion of people positive for yersinosis presenting to general practice in the second intestinal infectious disease study (IID2) in 2008 was lower than in the first study in 1995 (0.1% vs 1.8%), although different methodologies may limit direct comparison [13,14]. It has been hypothesised that *Yersinia* cases decreased during the 2000s due to the impact of the foot-and-mouth disease outbreaks on pork consumption, and better slaughterhouse hygiene [15]. Recent data from the South East region, however, indicate that yersiniosis in England may be substantially under-estimated, and that the presumed decline may be artefactual, at least in part. Our study suggests that the true incidence of *Y. enterocolitica* infections in England may be as high as, or higher than in other parts of Europe, the historical decline was artefactual, and that we may lack good data to identify the true trend over time. Extrapolation from the one laboratory reporting the results of PCR testing for *Yersinia* estimates that 7,500 infections may be undiagnosed nationally, although this is based on extrapolation of only one area and incidence may vary between regions and years.

We speculate that the markedly varying incidence across Europe may in part also reflect differential testing practice and substantial under-ascertainment in many other countries. Only between 6,000 and 7,000 confirmed cases in total have been reported in the European Union annually in recent years with an overall incidence of only 1.8 cases per 100,000 population in 2020 and striking variation in reported incidence between different countries [1]. In New Zealand, where the vast majority of faecal samples are tested using culture-independent diagnostic methods, the incidence in 2019 reached 24.1 cases per 100,000 population. The incidence of yersiniosis in New Zealand, however, was also high in the past. This could be partially explained by their long-standing practice to routinely test all diagnostic faecal samples for *Yersinia*, even before the introduction of culture-independent diagnostic testing [16].

Alongside uncertain overall yersiniosis incidence in England, there was a noticeable change in the age distribution of cases over time. The relative decrease in the proportion of cases in young children and an increase in the proportion of cases affecting people 65 years and older, far exceeding the rise in that age group as a proportion of the population, are striking. This could reflect changing testing practices or a real change in epidemiology. Similar patterns in areas with high reported incidence and other parts of England in each time period suggest that this change is real. In addition, almost one third of all cases in England in the most recent time period were diagnosed in May and June, while no such seasonal pattern was present in earlier data or in the rest of Europe [1]. There were no known outbreaks or changes in laboratory practices that would have affected the seasonal distribution of cases in the period from 2017 to 2020. Several real but unnoticed changes may thus have been occurring to the epidemiology of this infection in recent decades, at least in England.

The strengths of this study include the use of national laboratory surveillance data over five decades. Information on the patients’ region of residence and diagnostic laboratory allowed investigation of trends in different regions and laboratories over time and estimation of incidences in these regions and hospital catchment populations. We also involved the public in planning our research on *Yersinia* infections by discussing it with a Public and Patient Involvement group consisting of seven members of the public from a range of backgrounds advising on the work of the Health Protection Research Unit in Gastrointestinal Infections. They felt it was important to better understand the true burden of *Yersinia* infections in England, sources of infection and reasons for the lower number of cases when compared with international figures.

A weakness of this study is that only positive cases are reported to the national surveillance system with no information on the volume of testing. Testing and reporting practices may have changed substantially and it is likely that there is a variation in the interpretation of UK SMIs by different laboratories and what constitutes a clinical suspicion warranting testing for *Yersinia* [7], and perhaps variation in reporting practices. We were unable to accurately separate the impact of changing policy and guidelines on testing in England from changing epidemiology.

Similarly, testing protocols may have altered sensitivity. Most commercial PCR assays for gastrointestinal pathogens target *Y. enterocolitica* because other *Yersinia* species, such as *Y. pseudotuberculosis*, are relatively rare and less commonly associated with gastrointestinal symptoms [17]. The pathogenic potential of some of the other *Yersinia* species, including *Y. frederiksenii*, *Y. intermedia*, *Y. mollaretii*, *Y. bercovieri* and *Y. rohdei*, has been debated in the scientific literature [18,19]. We have focussed mainly on comparisons of *Y. enterocolitica* to reduce the impact of this. In addition, the national SGSS dataset does not include information on the serotypes and biotypes of *Yersinia* isolates. Previous analysis of human isolates submitted to the national Gastrointestinal Bacteria Reference Unit in England suggests a diversity of serotypes and biotypes, and although the most common biotype was 1A [20] which was previously not considered pathogenic, evidence is emerging that some strains of 1A can cause gastrointestinal disease [21,22]. The SGSS data we analysed also do not include information about the clinical presentation, severity and outcomes of *Yersinia* infections. Results from enhanced surveillance questionnaires suggest that yersiniosis may present a considerable burden to patients, with most reporting ongoing symptoms 2 weeks after disease onset. Only a minority of patients, however, completed the questionnaires, and they may not be fully representative of all patients. Over a third of patients who responded to the questionnaire had a bowel disease, and some of the *Yersinia* findings may have been incidental.

## Conclusion

Even accepting the limitations of our data we argue that our review of available data constitutes good evidence that (i) *Yersinia* infection is substantially under-reported in England, (ii) there have been real changes to the epidemiology of this infection that have gone unnoticed and (iii) there is a compelling argument for a structured approach to *Yersinia* surveillance in England and other countries that includes routine testing and follow-up of cases by epidemiological questionnaire. This might take the form of sentinel rather than universal surveillance but should include routine testing of available diarrhoeic samples in the populations surveyed. The advent of multiplex PCR tests for gastrointestinal infections including *Yersinia* will facilitate this. Culture and genomic analysis of positive samples may provide further insight into the source and transmission of *Yersinia* infections in England and support international comparison.
